# No evidence for human papillomavirus having a causal role in salivary gland tumors

**DOI:** 10.1186/s13000-018-0721-0

**Published:** 2018-07-18

**Authors:** Linnea Haeggblom, Ramona Gabriela Ursu, Leila Mirzaie, Tove Attoff, Caroline Gahm, Lalle Hammarstedt Nordenvall, Anders Näsman

**Affiliations:** 1 Department of Oncology-Pathology, Karolinska Institute, Cancer Center Karolinska, R8:01, Karolinska University Hospital, 171 76 Stockholm, Sweden; 2 Department of Microbiology, University of Medicine and Pharmacy Grigore T. Popa Iasi, 700115 Iasi, Romania; 3 Department of Oto-Rhino-Laryngology, Head and Neck Surgery, Karolinska University Hospital, 171 76 Stockholm, Sweden; 4 Department of Clinical Science, Intervention and Technology, Karolinska Institute, 171 76 Stockholm, Sweden; 5 Department of Clinical Pathology, Karolinska University Hospital, 171 76 Stockholm, Sweden

**Keywords:** Salivary gland tumors, Salivary gland malignancies, Human papilloma virus, HPV, Tumor viruses

## Abstract

**Background:**

Salivary gland malignancies are a very heterogeneous group of cancers, with histologically > 20 different subtypes, and prognosis varies greatly. Their etiology is unknown, however, a few small studies show presence of human papillomavirus (HPV) in some subtypes, although the evidence for HPV having a causal role is weak. The aim of this study was to investigate if HPV plays a causal role in the development of different parotid salivary gland tumor subtypes.

**Methods:**

DNA was extracted from 107 parotid salivary gland formalin fixed paraffin embedded tumors and 10 corresponding metastases, and tested for 27 different HPV types using a multiplex bead based assay. HPV DNA positive tumors were stained for p16^INK4a^ overexpression by immunohistochemistry.

**Results:**

One of the 107 malignant parotid salivary gland tumors (0.93%) and its corresponding metastasis on the neck were positive for HPV16 DNA, and both also overexpressed p16^INK4a^. The HPV positive primary tumor was a squamous cell carcinoma; neither mucoepidermoid nor adenoid cystic tumors were found HPV positive.

**Conclusions:**

In conclusion, HPV DNA analysis in a large number of malignant parotid salivary gland tumors, including 12 different subtypes, did not show any strong indications that tested HPV types have a causal role in the studied salivary gland tumor types.

**Electronic supplementary material:**

The online version of this article (10.1186/s13000-018-0721-0) contains supplementary material, which is available to authorized users.

## Background

Salivary gland malignancies are a very heterogeneous group of cancers, where histologically more than 20 different subtypes have been identified and prognosis varies greatly [1]. These tumors are rare, yet affect patients in all age groups and are mainly found in the parotid gland, but can also be found in submandibular, sublingual and accessory parotid glands [2, 3]. In Sweden about 100 new cases of salivary gland cancers are diagnosed each year, accounting for about 8% of head and neck cancers [3], which is similar to 6% of head and neck cancers in the United States [2]. Today the determination of tumor subtype is mainly based on the pathologist’s histological evaluation, and due to the large histological variability in this group of tumors, diagnoses are often determined with uncertainty. As a supportive measure genetic profiling is used in some cases, however, the sensitivity of the tests varies greatly. Moreover, it may often be with great uncertainty the prognostic tumor grade is determined, which is directly correlated to the treatment strategy. The most common treatment strategy is surgery, sometimes followed by postoperative radiation therapy [2, 3].

It has been well established by us and others that human papillomavirus (HPV) plays a causative role in a large proportion of oropharyngeal squamous cell carcinomas (OPSCC) [4–6] and that the mutational burden is lower in HPV positive OPSCC as compared to HPV negative OPSCC [7]. It is also well established that HPV can be valuable as a prognostic tool, since patients with HPV positive OPSCC have a much better clinical outcome compared to patients with HPV negative OPSCC [8]. However, whether HPV has a causal effect in salivary gland carcinomas is not as certain. A recent study indicated that there are major differences in mutation frequencies between different salivary gland tumor subtypes, where none or few mutations were found in mucoepidermoid, adenoid cystic and acinic cell subtypes [9], suggesting that other factors such as e.g. a viral infection may be the causative factor. There are a handful small studies that have shown an association between HPV infection and certain salivary gland malignancies, however, reported HPV prevalence varied to a great extent, as summarized in Additional file 1: Table S1 [10–17]. Moreover, these studies included only a small sample size, and most studies only included one or few specific subtypes of salivary gland tumors with very few samples in each subgroup, thus missing proper control groups. In this study we investigated the occurrence of HPV (defined as being HPV DNA positive and overexpressing p16^INK4a^) in, to our knowledge, the largest cohort of parotid salivary gland tumors, including 12 different subtypes, in patients diagnosed between 2000-2009 in the counties of Stockholm and Gotland.

## Methods

### Patients and tumor samples

Between 2000-2009, 145 patients in the counties of Stockholm and Gotland were identified and diagnosed with the following types of malignant parotid salivary gland tumors: acinic cell carcinoma, adenocarcinoma not otherwise specified (NOS), adenoid cystic carcinoma, basal cell adenocarcinoma, epithelial- myoepithelial carcinoma, mucoepidermoid carcinoma, myoepithelial carcinoma, oncocytic carcinoma, poorly differentiated carcinoma, salivary duct carcinoma, secretory carcinoma and squamous cell carcinoma (ICD-10: C07.9). From these, haematoxylin and eosin stained tumor sections were histologically evaluated to confirm presence of malignant cells and diagnosis. In total 117 biopsies (including 10 corresponding metastases) from 107 unique patients had sufficient tumor material for inclusion in this study and were collected as formalin fixed paraffin embedded (FFPE) tumors from the Clinical Pathology Department at Karolinska University Hospital. Sections from the FFPE tumors with, when possible, > 70% tumor cell area were taken for DNA extraction and immunohistochemistry (IHC) staining.

### DNA extraction and detection of HPV

DNA was extracted from 10 μm FFPE tumor tissue sections using the QIAamp DNA FFPE Tissue Kit (Qiagen, Hilden, Germany) following the manufacturer’s suggested protocol. In short, the FFPE sections were dissolved in Xylene followed by rehydration in absolute ethanol. After protein degradation the samples were purified in several steps using spin columns, thereafter the purified DNA was stored at − 20 °C. For each tumor sample a blank paraffin section was included as a negative control in order to detect possible cross-contamination between samples. Presence of HPV DNA was detected, following a 40 cycle PCR amplification using the QIAGEN multiplex PCR kit (Qiagen, Hilden, Germany) together with broad-spectrum GP5+/6+ primers (Cybergene, Stockholm, Sweden) [18]. PCR products were then hybridized to specific probes for 27 different HPV types (HPV 6, 11, 16, 18, 26, 30, 31, 33, 35, 39, 42, 43, 44, 45, 51, 52, 53, 56, 58, 59, 66, 67, 68, 69, 70, 73 and 82) and evaluated on a Magpix instrument (Luminex Corporation, Austin, USA) for the different HPV types as previously described [19, 20]. β-globin was included as a positive control for presence of DNA as described previously [21]. In addition, negative controls with distilled water and positive controls with SiHa cells corresponding to 5, 50 and 500 HPV genomes were also included and treated per protocol.

### p16^INK4a^ overexpression

The tumor that tested positive for HPV was also evaluated for p16^INK4a^ tumor suppressor protein (p16) overexpression. p16 overexpression was determined by IHC using the mouse monoclonal antibody CINtec® p16 Histology (clone E6H4, Ventana, Roche Diagnostics, Basel, Switzerland), as previously described [21]. The sections were evaluated by a pathologist, where a ≥ 70% stained tumor was considered as positive for p16 overexpression, and < 70% as negative.

### Statistical analysis

Fisher’s exact test was used to compare HPV numbers per sub-group of salivary gland tumors.

## Results

In total 117 tumor samples, including 10 corresponding metastases, from 12 different salivary gland tumor types, were analyzed for presence of HPV DNA (Table 1). All samples were positive for the β-globin gene used as a positive control, indicating successful DNA purification. One out of the 107 (0.93%) tested malignant salivary gland primary tumors was positive for HPV16, which was 1/7 salivary gland squamous cell carcinomas included in this study. This tumor also showed p16 overexpression. No significant overrepresentation of HPV DNA was observed in squamous cell carcinomas as compared to the other subgroups (*p* = 0.06, Table 1). Moreover, the HPV16 and p16 positive tumor had a corresponding metastasis located on the neck, which also was positive for HPV16 and p16.

**Table 1:** Patient and tumor characteristics

Histological subtype^a^	Tumors (N)	Age (mean)	TNM-stage^b^									Available regional metastasis	HPV DNA+ primary tumors^c^	HPV DNA+ regional metastasis^c^
			T				N			M				
			T1	T2	T3	T4	N0	N1	N2	M0	M1			
Acinic cell carcinoma	22	54	9	10	2	1	22	0	0	22	0	–	0	–
Adenocarcinoma, UNS	19	66	3	8	2	6	13	2	4	17	2	4/6	0	0
Adenoid cystic carcinoma	13	55	5	6	1	1	13	0	0	1	2	–	0	–
Basal cell adenocarcinoma	3	75	2	0	1	0	3	0	0	3	0	–	0	–
Epithelial-myoepithelial carcinoma	4	75	1	2	1	0	4	0	0	4	0	–	0	–
Mucoepidermoid carcinoma	22	47	11	9	0	2	21	1	0	21	1	0/1	0	0
Myoepithelial carcinoma	2	53	0	2	0	0	1	0	1	1	1	1/1	0	0
Oncocytic carcinoma	1	65	0	1	0	0	1	0	0	1	0	–	0	–
Poorly differentiated carcinoma	3	85	0	1	1	1	1	1	1	3	0	1/2	0	0
Salivary duct carcinoma	10	57	2	3	1	4	4	2	4	9	1	2/6	0	0
Secretory carcinoma	1	85	1	0	0	0	1	0	0	1	0	–	0	–
Squamous cell carcinoma	7	56	2	2	3	0	4	1	2	7	0	2/3	1^d, e^	1^d^
Total	107	59										10	1	1

^a^Histological subtype according to WHO 2017

^b^TNM-stage according to AJCC 7th Ed

^c^Human papillomavirus DNA positivity (HPV DNA+) as assessed by presence of HPV DNA by Luminex Multiplex PCR

^d^Primary tumor and regional metastasis obtained from the same patient

^e^Fischer’s exact test for HPV positivity in squamous cell carcinoma vs. all other sub-types: *p* = 0.06

The patient with an HPV positive tumor was a 78-year old male patient with severe co-morbidities and no history of other tumors. Tumor origin was evaluated clinically and radiologically with no evidences of other origins than the parotid gland; the oropharynx was however not assessed histologically. Since the patient died some months after diagnosis, the follow-up was limited.

## Discussion

In this study, 107 malignant salivary gland tumors, including 12 different subtypes, with the addition of 10 corresponding metastases were tested for the presence of 27 different HPV types (including all known high risk HPV types) using a multiplex bead based assay. One of the 107 malignant salivary gland tumors and its corresponding metastasis on the neck were positive for HPV16 DNA, and both also overexpressed p16 and therefore could be defined as being HPV positive. All other subtypes tested were HPV DNA negative. In total, only one of seven squamous cell carcinoma salivary gland tumors was HPV positive and due to the small sample size of squamous cell salivary gland tumors it was not possible to draw any reliable conclusion to whether HPV was involved in the development of this tumor type. Furthermore, all other subgroups lacked high risk HPV DNA and did not express presence of any of the other tested HPV types, suggesting that HPV does not play a major role as a causative agent in these subgroups. The latter was shown in a more limited study by Skalova et al. [13].

Nonetheless, HPV positive head and neck cancers often present with an HPV positive neck metastasis, and it has been shown that HPV positive cancer of unknown primary of the head and neck region often originate from an HPV positive OPSCC [22, 23]. Hence there is also a possibility that the HPV16 positive squamous cell salivary gland carcinoma found in this study was instead a primary tumor, or a cancer of unknown primary originating from an undetected OPSCC. This would further support that HPV does not play a major role for the development of salivary gland tumors. However, the clinical evaluation in this case did not support such an assumption.

There are some limitations to this study. Firstly, salivary gland tumors are a rare and heterogeneous group of tumors including more than 20 different subtypes, making it difficult to gather representable amounts of material for this type of analysis. Furthermore, although, to our knowledge, the presence of HPV was analyzed in the largest cohort published so far, the number of samples per subgroup was still limited, which makes it difficult to draw any clear conclusions. Secondly, the presence of p16 overexpression in samples was only tested on the one sample, and its corresponding metastasis, that both had tested positive for HPV DNA. Nevertheless, previous studies have shown p16 overexpression in malignant salivary gland tumor cohorts, still lacking or having a low correlation to presence of HPV [11, 13]. These findings may also be indicative of HPV not playing the cancer-driving role and that p16 in these tumors is a bad surrogate marker for an active HPV infection. Lastly, the HPV16 positive tumor found in this study was one out of seven squamous cell carcinomas, which is a very rare type of salivary gland tumor. Here, no significant overrepresentation of HPV was observed in squamous cell carcinomas as compared to the other sybtypes. However, the numbers of squamous cell carcinomas was restricted and the results should be interpreted with caution. With a larger cohort of squamous cell salivary gland carcinomas, perhaps more conclusive results could be drawn to whether HPV is a causative agent in the development of these tumors or not.

## Conclusions

In conclusion, HPV DNA analysis in a large number of malignant salivary gland tumors, including 12 different subtypes, did not show any strong indications that tested HPV types have a causal role in the studied salivary gland tumor types.

## Additional file

Additional file 1: **Table S1.** Summary of studies on salivary gland tumors in relation to HPV infection as referred to in the background section. (DOCX 33 kb)

## Abbreviations

FFPE: Formalin fixed paraffin embedded
HPV: Human papillomavirus
IHC: Immunohistochemistry
NOS: Not otherwise specified
OPSCC: Oropharyngeal squamous cell carcinoma
p16: p16^INK4a^ tumor suppressor protein
