# Supplementary material for: No evidence for human papillomavirus having a causal role in salivary gland tumors
Source: Diagn Pathol. 2018 Jul 18;13:44. doi: 10.1186/s13000-018-0721-0 (PMC6052678; doi:10.1186/s13000-018-0721-0)
Supplement: Supplementary file 1 — Table S1. Summary of studies on salivary gland tumors in relation to HPV infection as referred to in the background section. (DOCX 33 kb) [file 13000_2018_721_MOESM1_ESM.docx]

Supplementary Table 1.

| Publication | Country | Samples size | % HPV DNA+ tumors | % p16+ tumors | Tumor subtypes/sites included | HPV+ tumor subtypes |
| --- | --- | --- | --- | --- | --- | --- |
| Boland et al. [11] | USA | 27 | 7.4% | 11% (>75% tumor cells stained) | Adenoid cycstic carcinoma from:  maxilla  submandibular gland  sublingual gland  soft Palate  sphenoid/nasal septum  base of tongue  palate  external auditory canal  parotid gland  sphenoid/ethmoid  lacrimal gland  larynx  nasal cavity | adenoid cystic carcinoma from:  nasal Cavity |
| Vageli et al. [12] | Greece | 9 | 78% | - | oncocytoma  acinic cell carcinoma  high-grade adenocarcinoma  low-grade polymorphous adenocarcinoma  Warthin's tumors  pleomorphic adenomas  lymphoepithelial cysts  lipoma of the parotid gland | oncocytoma  pleomorphic adenomas  Warthin's tumors  (infected with high risk HPVs) |
| Skálová et al. [13] | Czech Republic | 55 | - | 9.1% | Benign salivary gland tumors:  pleomorphic adenoma  Warthin tumor  myoepithelioma  oncocytoma  cystadenoma  basal cell adenoma  Malignant salivary gland tumors:  polymorphous low grade adenocarcinoma  cribriform adenocarcinoma of tongue  adenoid cystic carcinoma of minor glands  salivary duct carcinoma  mucoepidermoid carcinoma  acinic cell carcinoma  hyalinizing clear cell carcinoma  carcinosarcoma  pleomorphic adenoma | - |
| Hafed et al. [14] | Egypt | 34 | 24% | - | Benign salivary gland neoplasms:  pleomorphic adenoma  myoepithelioma  Warthin's tumour  Malignant salivary gland neoplasms:  adenoid cystic carcinoma  epimyoepithelial carcinoma  lymphoepithelial carcinoma  lymphoma  mucoepidermoid carcinoma  myoepithelial carcinoma  polymorphous low grade adenocarcinoma. | Benign salivary gland neoplasms:  Warthin's tumour pleomorphic adenoma myoepithelioma  Malignant salivary gland neoplasm:  lymphoma |
| Lin et al. [15] | Taiwan | 53 | 36% | - | pleomorphic adenoma  Warthin’s tumor  basal cell adenoma  mucoepidermoid carcinoma  myoepithelial tumor | pleomorphic adenoma  Warthin’s tumor  mucoepidermoid carcinoma |
| Qian et al. [16] | Germany | 67 | 42% | 58% | adenoid cycstic carcinoma:  parotid  submandibular  minor salivary gland | adenoid cycstic carcinoma:  parotid  submandibular  minor salivary gland |
| Xu et al. [17] | USA | 8 | 36% | 50% | primary parotid squamous cell carcinoma | primary parotid squamous cell carcinoma |

11. Boland JM, McPhail ED, García JJ, Lewis JE, Schembri-Wismayer DJ. Detection of human papilloma virus and p16 expression in high-grade adenoid cystic carcinoma of the head and neck. Mod Pathol. 2012;25:529–36. doi:10.1038/modpathol.2011.186.

12. Vageli D, Sourvinos G, Ioannou M, Koukoulis GK, Spandidos DA. High-risk human papillomavirus (HPV) in parotid lesions. Int J Biol Markers. 2007;22:239–44.

13. Skalova A, Kaspirkova J, Andrle P, Hosticka L, Vanecek T. Human papillomaviruses are not involved in the etiopathogenesis of salivary gland tumors. Ces Patol. 2013;49:72–5.

14. Hafed L, Farag H, Shaker O, El-Rouby D. Is human papilloma virus associated with salivary gland neoplasms? An in situ-hybridization study. Arch Oral Biol. 2012;57:1194–9.

15. Lin FC, Chen PL, Tsao TY, Li CR, Jeng KC, Tsai SC. Prevalence of human papillomavirus and Epstein-Barr virus in salivary gland diseases. J Int Med Res. 2014;42:1093–101.

16. Qian X, Kaufmann AM, Chen C, Tzamalis G, Hofmann VM, Keilholz U, et al. Prevalence and associated survival of high-risk HPVrelated adenoid cystic carcinoma of the salivary glands. Int J Oncol. 2016;49:803–11. doi:10.3892/ijo.2016.3563.

17. Xu B, Wang L, Borsu L, Ghossein R, Katabi N, Ganly I, et al. A proportion of primary squamous cell carcinomas of the parotid gland harbour high-risk human papillomavirus. Histopathology. 2016;:921–9.

Summary of studies on salivary gland tumors in relation to HPV infection as referred to in the background section.
